# Case report: Sacral agenesis in two boxer dogs: clinical presentation, diagnostic investigations, and outcome

**DOI:** 10.3389/fvets.2023.1201484

**Published:** 2023-05-25

**Authors:** Diletta Dell'Apa, Martina Fumeo, Antonella Volta, Marco Bernardini, Francesca Fidanzio, Valentina Buffagni, Matthias Christen, Vidhya Jagannathan, Tosso Leeb, Ezio Bianchi

**Affiliations:** ^1^Department of Veterinary Science, University of Parma, Parma, Italy; ^2^Neurodiagnostic Unit, Anicura Portoni Rossi Veterinary Hospital, Bologna, Italy; ^3^Department of Animal Medicine, Production and Health, Clinical Section, University of Padua, Legnaro, Italy; ^4^Vetsuisse Faculty, Institute of Genetics, University of Bern, Bern, Switzerland

**Keywords:** sacral agenesis, sacro-caudal dysgenesis, Currarino syndrome, spina bifida, congenital spine malformation, caudal regression syndrome

## Abstract

Two boxer dogs from the same litter were presented at 3 months of age for urinary and fecal incontinence. Both dogs had an abnormal tail consisting of a small stump, an atonic anal sphincter, and absent perineal reflex and sensation. Neurological evaluation was indicative of a lesion of the cauda equina or sacral spinal cord. Radiology and CT scan of the spine displayed similar findings in the two dogs that were indicative of sacral agenesis. Indeed, they had 6 lumbar vertebrae followed by a lumbosacral transitional vertebra, lacking a complete spinous process, and a hypoplastic vertebra carrying 2 hypoplastic sacral transverse processes as the only remnant of the sacral bone. Caudal vertebrae were absent in one of the dogs. On MRI, one dog had a dural sac occupying the entire spinal canal and ending in a subfascial fat structure. In the other dog, the dural sac finished in an extracanalar, subfascial, well-defined cystic structure, communicating with the subarachnoid space, and consistent with a meningocele. Sacral agenesis—that is the partial or complete absence of the sacral bones—is a neural tube defect occasionally reported in humans with spina bifida occulta. Sacral agenesis has been described in human and veterinary medicine in association with conditions such as caudal regression syndrome, perosomus elumbis, and Currarino syndrome. These neural tube defects are caused by genetic and/or environmental factors. Despite thorough genetic investigation, no candidate variants in genes with known functional impact on bone development or sacral development could be found in the affected dogs. To the best of the authors' knowledge, this is the first report describing similar sacral agenesis in two related boxer dogs.

## 1. Introduction

Neural tube defects (NTD) are the most common human birth defects. In both humans and animals, these malformations can be caused by genetic or environmental factors. Genetic factors account for approximately 70% of human cases (1). Other non-genetic factors involved in the abnormal closure of the neural tube include drugs (e.g., valproic acid) (2), environmental teratogens (industrial waste and pesticides) (3), and maternal factors like obesity, folic acid deficiency, diabetes mellitus, and hyperthermia during the first trimester of pregnancy (1, 4).

NTDs are usually divided into open and closed defects. The open defects are characterized by external protrusion and/or exposure of neural tissue, while in closed defects the neural tissue is covered by other tissue (4).

Spina bifida (SB) is an NTD caused by incomplete caudal neurulation (4). It results in the failure of fusion of one or more vertebral arches with or without protrusion of the meninges and the spinal cord. This malformation, with hemivertebrae and transitional vertebrae, frequently occurs in dogs with screw tails. Vertebral malformations do not necessarily produce neurological signs and are often seen in radiographs as incidental findings (5).

In humans, sacro-caudal dysgenesis and sacral agenesis (SA) are rarely described NTDs. These terms are used to identify a spectrum of malformations of the sacrum and caudal vertebrae of varying severity. In particular, the term SA identifies the partial or complete absence of the sacral bones. In these syndromes, the most frequent neurological signs are urinary and fecal incontinence, sometimes accompanied by hind limb paresis or paralysis (5).

In small animals, similar malformations have previously been reported in Manx cats and occasionally in dogs (6, 7). In Manx cats, sacro-caudal dysgenesis is well known and can also be associated with myelodysplasia, meningocele, meningomyelocele, tethered spinal cord, hydromyelia, and/or syringomyelia (8, 9). An autosomal dominant inheritance with incomplete penetrance has been demonstrated for this feline disease (7). The tailless phenotype and increased risk for sacro-caudal dysgenesis in Manx and other tailless cats is caused by variants in the *TBXT* gene encoding the T-box transcription factor T (10). Tailless or short-tailed phenotypes in several dog breeds are also caused by dominant alleles of the *TBXT* gene (11, 12).

Perosomus elumbis (PE) is a complex syndrome that has been commonly described in farm animals and more rarely in puppies. It is characterized by partial or complete agenesis of the lumbar, sacral, and coccygeal vertebra, and sometimes by other malformations involving the limbs, such as arthrogryposis (13, 14).

In this case report we describe the clinical signs, imaging, and electrodiagnostic findings and outcome of SA in two related boxer dogs. The results of genetic investigation are also reported.

## 2. Case description

Two 3-month-old intact female boxer dogs from the same litter were presented to the Veterinary Teaching Hospital (VTH) of the University of Parma for the presence of urinary and fecal incontinence since birth. The other four littermates and the parents did not present similar clinical signs.

On presentation, physical examination was unremarkable except for the tail, which appeared as a small stump in both dogs. Neurological examination revealed the presence in both dogs of an atonic anal sphincter and the absence of perineal reflex and nociception. No signs of paraparesis or sciatic nerve involvement were present. Hyperestesia of the lumbosacral spine was not detected. A cauda equina or sacral spinal cord lesion was suspected based on neurological examination. Differential diagnoses included, in particular, anomalous diseases such as SB associated with meningocele or meningomyelocele, sacral dysgenesis/agenesis, and other spine malformations.

**Figure 1:**
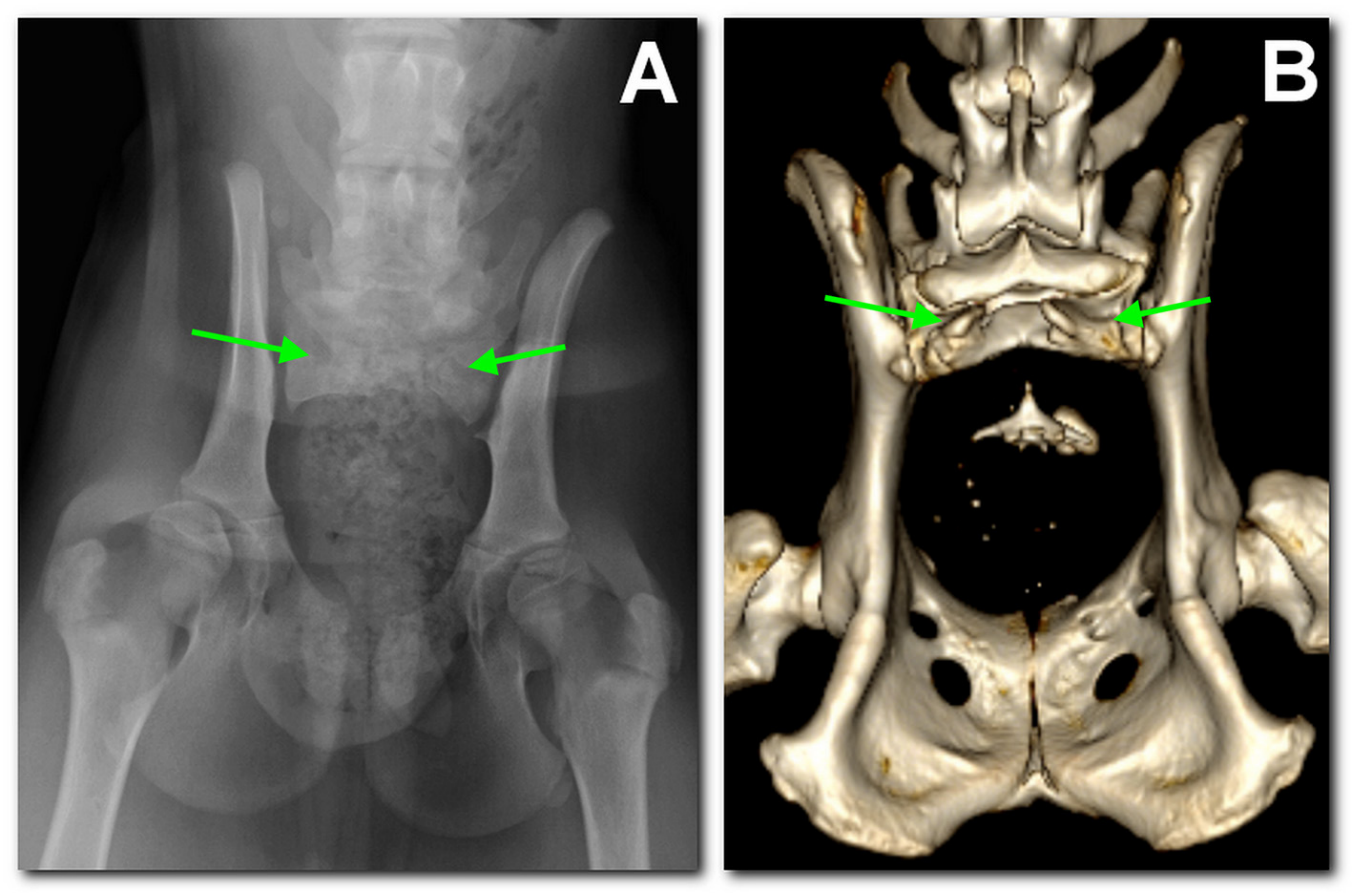
Dog 1, radiographic study with a ventro-dorsal projection of the pelvis performed at 3 months of age **(A)** in comparison to the tomographic 3D volume rendering reconstruction of the pelvis at 18 months of age **(B)**. There is sacralization of L7. Only S1 is present and hypoplastic; the dorsal part of the arch is not fused and has no spinal process (between arrows). Caudally, there is a single coccygeal vertebra not articulating with the sacrum.

**Figure 2:**
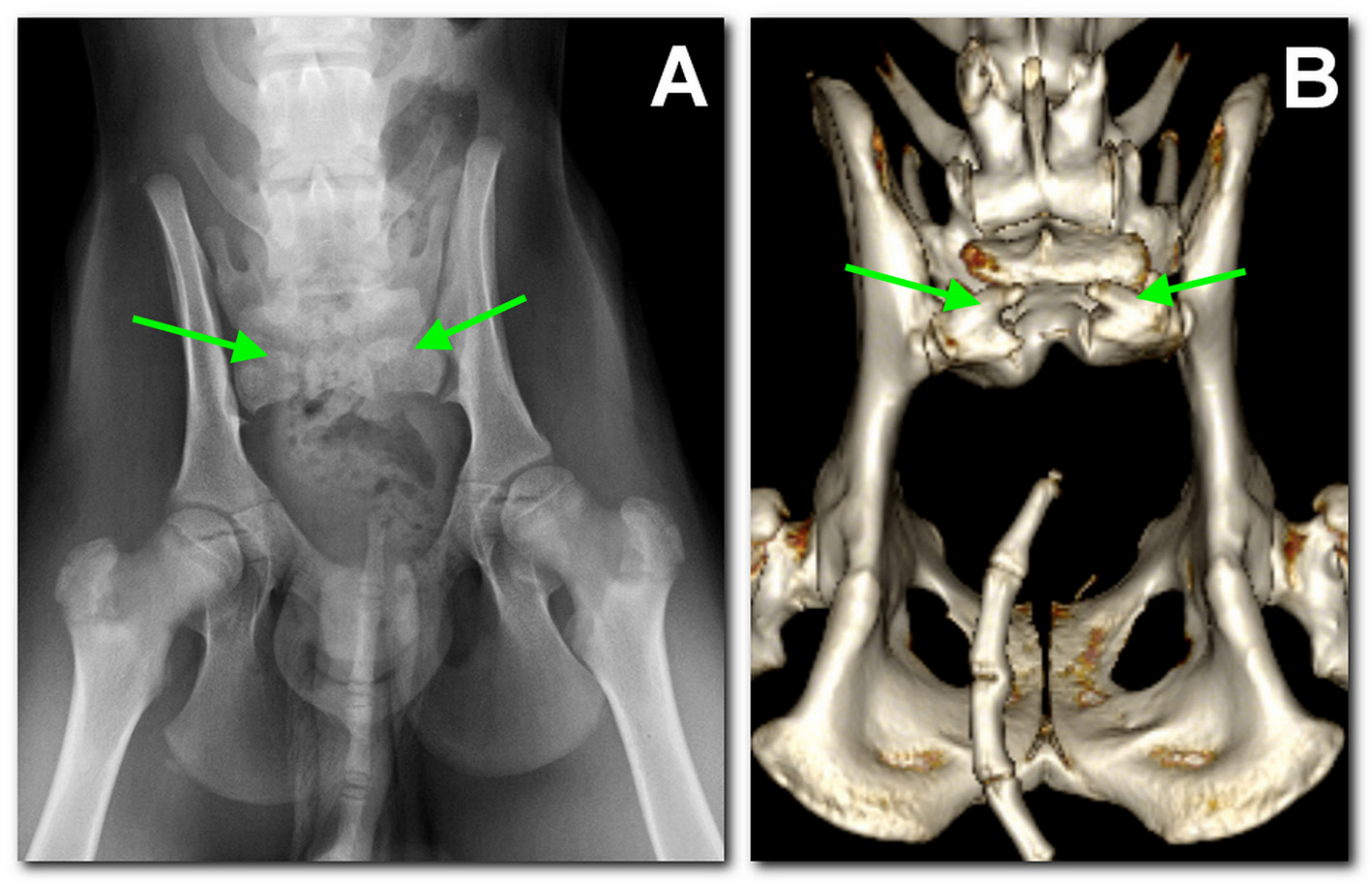
Dog 2, radiographic study with a ventro-dorsal projection of the pelvis performed at 3 months of age **(A)** in comparison to the tomographic 3D volume rendering reconstruction of the pelvis at 18 months of age **(B)**. Similar findings as in dog 1 are noted: L7 sacralization, S1 with spina bifida (between arrows), and sacral agenesis. Caudally, several coccygeal vertebrae are present, not articulating with S1.

A radiographic study of the lumbosacral spine was made in the two puppies in both lateral and ventro-dorsal views. Both patients showed six typically shaped lumbar vertebrae, followed by a lumbosacral transitional vertebra lacking the spinous process and a hypoplastic vertebra carrying two hypoplastic sacral transverse processes as the only remnant of the sacral bone. These findings were considered compatible with SA. Caudal vertebrae were absent in dog 1 (Figure 1A), while in dog 2 several coccygeal vertebrae not articulating with the rest of the spine were present (Figure 2A).

**Figure 3:**
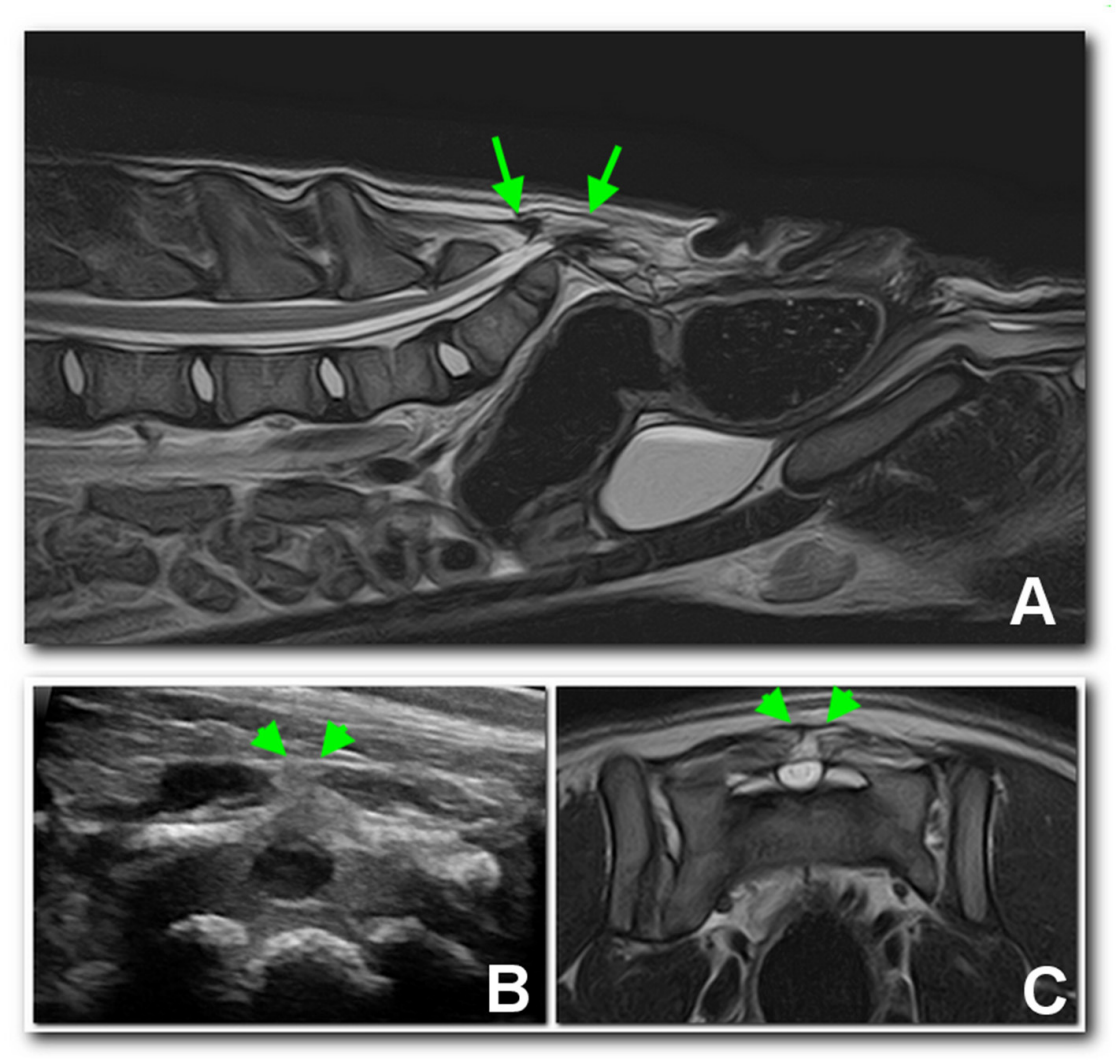
Sagittal **(A)** and transverse **(C)** T2 MRI images of the lumbosacral region of dog 1, compared with an ultrasonographic transverse scan **(B)** of the dorsal sacral aspect. A dural sac occupying the entire spinal canal protruding dorsally (arrows) and ending in a subfascial fat structure may be noted between the arrowheads.

**Figure 4:**
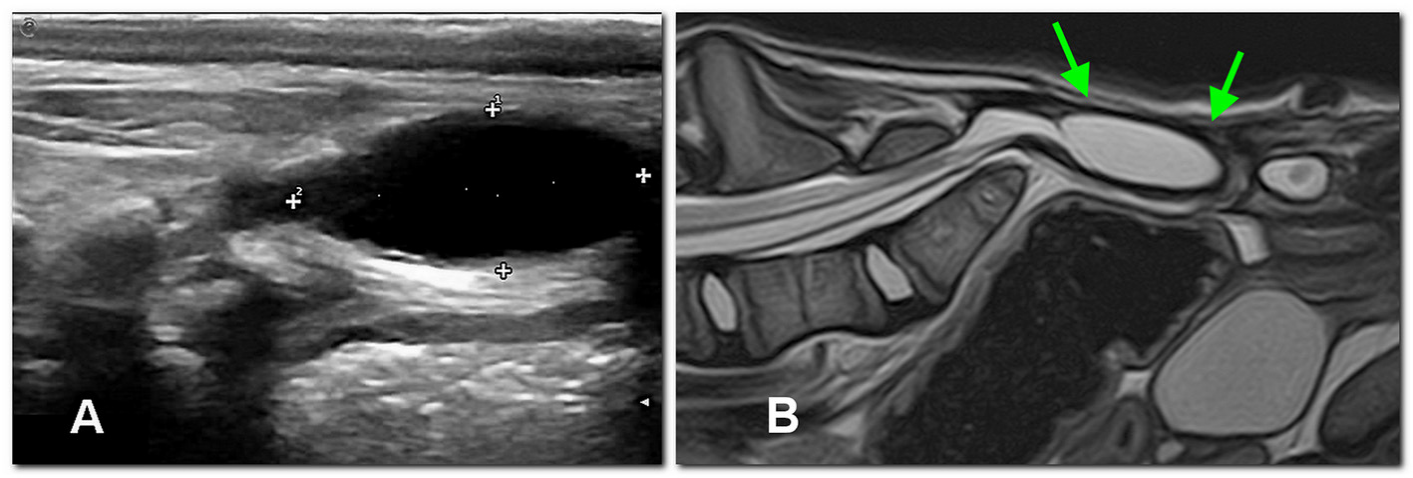
In dog 2, an extracanalar, well-defined subfascial cystic structure caudal to the spine, likely consisting of meningocele, is displayed between calipers by the ultrasonographic sagittal study **(A)** at 3 months of age and later confirmed by MRI [sagittal T2 image, **(B)**] at 4 months of age (arrows).

Ultrasound examination of the dorsal sacral region was carried out using a microconvex probe (6–10 MHz). In both patients, the spinal cord was visible, partially protruded caudally, due to the incomplete fusion of the dorsal lamina (Figure 3B). In dog 2, a well-defined anechoic oval 2 x 0.8 cm structure consistent with a meningocele was observed (Figure 4A).

At 4 months of age, complete blood count and blood chemistry values were unremarkable. In view of eventual surgical planning, an MRI (1.5 T, Vantage Elan, Canon Medical Systems Europe B.V., Zoetermeer, The Netherlands) of the lumbosacral spine was performed under general anesthesia with the dogs in dorsal recumbency. The imaging protocol included sagittal and transverse fast spin echo (FSE) T2-weighted (T2W) images [repetition time (ms) (TR)/echo time (ms) (TE), 3886–6353/108]; sagittal and transverse FSE T1-weighted (T1W) images (TR/TE, 480-745/14-15); transverse short tau inversion recovery (STIR) (TR/TE, 7139/48); 3D T1W fat suppressed gradient echo (GE) images (TR/TE, 32.2/5.5). No contrast medium was used. Besides the osteoarticular abnormalities in the radiographs, in dog 1, a dural sac occupying the entire spinal canal and ending abruptly in a subfascial, somewhat organized fat structure was observed (Figures 3A–C). In dog 2, the dural sac occupied the entire spinal canal and ended in a well-defined extracanalar, subfascial cystic structure, caudal to the spine and likely communicating with the subarachnoid space, and probably consisting of a meningocele, thus confirming ultrasound findings (Figure 4B). Surgical treatment of the meningocele was proposed but was declined by the owner. The dogs were discharged without medication, with the indication to monitor urination and defecation.

In the following months, urinary and fecal incontinence persisted without any other major symptoms. On the occasion of ovariohysterectomy, at 18 months of age, a computed tomography (CT) scan was performed as a follow-up. CT examination confirmed and better defined the skeletal malformations previously detected. In particular, both dogs presented L7 sacralization and partial sacral agenesis, consisting of the presence of a vestigial S1, and presenting a severely incomplete body that was not dorsally fused and had no spinous process, likely compatible with a SB (Figures 1B, 2B). The dural sac protruded caudally and dorsally in the subfascial fat, slightly in dog 1, while in dog 2 it formed a 1.3-cm oval hypoattenuating pouch compatible with meningocele. Needle electromyography (EMG) performed following CT scan revealed the presence of spontaneous pathological activity in the perineal muscles of both dogs, represented mainly by complex repetitive discharges.

To identify a potential causative genetic variant, whole genome 2 x 150 bp paired-end-sequencing at 20x coverage of one of the affected dogs was performed. The sequence data were deposited under study accession PRJEB16012 and sample accession SAMEA110415686 in the European Nucleotide Archive. The whole genome sequence data were compared to 924 control genomes (Supplementary Table 1), and variants particular to the boxer with sacral agenesis were identified as described (15). All identified private homozygous and private heterozygous variants are listed in Supplementary Table 2. None of these variants represented a strong plausible candidate for the sacral agenesis.

## 3. Discussion

In this case report we describe an NTD in two boxer dogs from the same litter. To precisely define the spinal anomaly present, the dogs underwent a complete diagnostic work-up that included various imaging modalities. In particular, radiology, ultrasonography, CT, and MRI performed at different stages of the dogs' growth were indicative of the presence of sacral agenesis (SA) associated with meningocele. Further, the incomplete S1 bone, which was not dorsally fused and had no spinous process, was compatible with an SB.

NTDs have a multifactorial etiology, with contributions from genetic, environmental and nutritional factors synergistically affecting normal embryonic development (3, 16).

During neurulation, the ectoderm grows in a longitudinal axis on the notochord and thickens to become a neural plate. Then, a neural groove appears as the edges of the neural plates become raised on each side of a midline depression, and, following the fusion of the edges in a bidirectional way, the neural tube is formed. In dogs, the closure starts in the cervical region and then progresses rostrally to form the future brain, and caudally to form the spinal cord. During this process, the ectoderm separates from the neural tube and fuses dorsally to create the overlying skin (17).

SB is an NTD reported in both humans and animals that results from the incomplete fusion of the dorsal lamina with or without protrusion of meninges (meningocele) or meninges and spinal cord (meningomyelocele). SB can also be classified as closed (occulta) or opened (manifesta/aperta) if there is communication between the neural tissue and the external environment. SA is occasionally reported in humans with SB occulta (5, 18–21).

SA with associated meningocele was initially identified in our patients at 3 months of age with radiographs and ultrasound of the lumbosacral spine. In human medicine, ultrasonography is also commonly used as a first-line screening test for spinal cord abnormalities in infants (9, 22). This rare malformation has previously occasionally been reported in dogs and in Manx cats (6, 7).

A more detailed characterization of SA and SB of the two subjects was possible in the following months with advanced imaging techniques (CT and MRI). Abnormalities similar to those detected in the two dogs have been reported in some rare syndromes described in humans and animals: Caudal regression syndrome (CRS), perosomus elumbis (PE), and Currarino syndrome (CS).

CRS is reported in humans, characterized by different degrees of anomaly of the caudal spinal cord and spine. In CRS there is the partial or total absence of the thoracic, lumbar, sacral, or coccygeal vertebrae associated with visceral organ malformations, but there is also imperforate anus and severe limb deformities. Diagnostic follow-up of suspicion can be made during pregnancy, detecting anomalies in the limbs and vertebral column with prenatal ultrasound. A prevalence of this congenital malformation has been identified in children of diabetic mothers, but other risk factor include cocaine and alcohol consumption. In children, walking disabilities are most commonly detected because of limb deformities (23–25).

SA is considered a part of CRS and can be classified into four types. In type I, there is partial or total unilateral agenesis localized to the sacrum; type II is characterized by partial bilaterally symmetrical agenesis with a firm articulation between the ilia and a normal or hypoplastic first sacral vertebra; in type III there is total sacral agenesis and a variable lumbar vertebra articulating with the ilia; and in type IV the sacrum is absent, while the caudal end plate of the lumbar vertebra is fused with the ilia. These anomalies are associated with a high prevalence of fecal incontinence and urinary symptoms (26, 27). Our patients display similarities with Type II SA.

SA is also a feature of CS, another syndrome reported in human medicine. Two forms of CS have been described: the classic form in which there is partial sacral agenesis, presacral mass (meningocele, lipoma, enteric cyst, teratoma, or a combination of these), and anorectal malformation, and the incomplete form in which only one or two of these malformations are present (28, 29). *HLBX9* gene variants have been reported in some CS patients (30–32). In the two boxer dogs included in the present study, anorectal malformation was absent, but presacral mass and SA were detected without other anomalies affecting the hind limbs.

PE is a rare complex congenital malformation that has been described in animals. It has been reported in only one dog, and one cat, and occasionally in cattle, buffalos, sheep, foals, pigs, rhesus macaques (*Macaca mulatta*), and it has been experimentally induced in mice (13, 33–43). As with CRS, animals with PE display agenesis of the caudal spinal cord and spine. Other signs associated with this syndrome are arthrogryposis and hypoplastic hind limbs, urogenital and intestinal tract defects, craniofacial malformation, synophthalmia, and brachygnatia. If the affected animal survives the first hours of life it usually displays severe difficulty in standing, due to the anomalies affecting the hind limbs and vertebra column. Euthanasia is usually performed, in light of the severity of this condition (34–36, 40).

In children, these congenital malformations require a multidisciplinary approach depending on the identified severity of anomalies. The diagnostic approach to fecal and urinary incontinence in patients with sacral anomalies is complex and involves various tests such as contrast colonography, colonic transit time, anorectal manometry, urodynamics, and EMG (44–46).

Unfortunately, tests for colonic function and urodynamic studies were not performed in these dogs. EMG in both dogs showed the presence of complex repetitive discharges of the perineal muscles as a possible consequence of chronic denervation. The absence of spontaneous pathologic activity at EMG of the muscles of the hind limbs further ruled out the presence of sciatic nerve involvement.

Early surgical treatment of meningocele/meningomyelocele associated with SB has been reported to improve neurological signs in some cases (18). Surgical treatment was declined by the owner, who considered the quality of life of the dogs to be good.

In the long-term follow-up (~3 years from the first examination), the two boxer dogs remained clinically stable and developed no major complications as a result of their urinary and fecal incontinence.

The occurrence of sacral dysgenesis in more than one puppy from a litter with healthy parents and an uneventful pregnancy suggested a genetic disorder with autosomal recessive inheritance. Alternatively, a *de novo* mutation event in the germline of one of the two parents might have generated a dominant allele causing the SA. Despite extensive genetic investigation, no candidate variants in genes with known functional impact on bone development or sacral development were found. One cautionary note is that short read whole genome sequencing does not have 100% sensitivity in detecting all genetic variants. In particular, large structural variants involving more than ~30 nucleotides are easily missed with the approach. Furthermore, gaps in the genome reference assembly or errors in the gene annotation of the dog genome might also explain why we were not able to identify a candidate for causative variant. The presence of environmental factors (toxic, nutritional, metabolic) as a possible cause of the malformation cannot be excluded.

Moreover, the concomitant presence of both genetic and non-genetic factors acting synergistically is also a possibility in the two boxer siblings. Indeed, in human medicine, the most reliable pathogenetic model for NTDs (*multifactorial threshold model*) considers either genetic and environmental factors on their own insufficient to affect neural tube closure. The concomitant presence of genetic and non-genetic factors is usually deemed necessary to induce NTDs (4).

To the best of the authors' knowledge, this is the first report of a similar SA in two related boxer dogs. Further studies including subjects with similar NTDs from this and other breeds should be performed in order to improve understanding of the role of genetic and non-genetic factors in these defects of neural tube closure.

## Data availability statement

The datasets presented in this study can be found in online repositories. The names of the repository/repositories and accession number(s) can be found below: https://www.ncbi.nlm.nih.gov/biosample; SAMEA110415686.

## Ethics statement

Ethical review and approval were not required for the animal study because the case report is a description of a clinical case. Written informed consent was obtained from the owners for the participation of their animals in this study.

## Author contributions

DD, EB, MF, AV, and FF contributed to management of the case. MF and AV were in charge of the CT scan, and MB of the MRI. MC, VJ, and TL performed the genetic testing. DD, VB, MF, MC, MB, AV, TL, and EB participated in the review and editing of the manuscript. All authors contributed to the article and approved the submitted version.

## References

[1] Finnell RH Caiaffa CD Kim SE Lei Y Steele J Cao X. Gene environment interactions in the etiology of neural tube defects. Front Genet. (2021) 12:659612. 10.3389/fgene.2021.659612 34040637 PMC8143787

[2] Wlodarczyk BJ Palacios AM George TM Finnell RH. Antiepileptic drugs and pregnancy outcomes. Am J Med Genet A. (2012) 158A:2071–90. 10.1002/ajmg.a.35438 22711424 PMC3402584

[3] Copp AJ Greene ND. Genetics and development of neural tube defects. J Pathol. (2010) 220:217–30. 10.1002/path.2643 19918803 PMC4239538

[4] Avagliano L Massa V George TM Qureshy S Bulfamante GP Finnell RH. Overview on neural tube defects: from development to physical characteristics. Birth Defects Res. (2019) 111:1455–67. 10.1002/bdr2.1380 30421543 PMC6511489

[5] Westworth DR Sturges BK. Congenital spinal malformations in small animals. Vet Clin North Am Small Anim Pract. (2010) 40:951–81. 10.1016/j.cvsm.2010.05.009 20732600

[6] Choi SY Lee I Cho NY Shin BH Lee KJ Choi HJ. Imaging diagnosis of sacrocaudal dysgenesis in a shih-tzu dog. J Vet Clin. (2016) 33:389–91. 10.17555/jvc.2016.12.33.6.389

[7] Deforest ME Basrur PK. Malformations and the Manx syndrome in cats. Can Vet J. (1979) 20:304–14. 393376 PMC1789620

[8] Leipold HW Huston K Blauch B Guffy MM. Congenital defects on the caudal vertebral column and spinal cord in Manx cats. J Am Vet Med Assoc. (1974) 164:520–3. 4813411

[9] Plummer SB Bunch SE Khoo LH Spaulding KA Kornegay JN. Tethered spinal cord and an intradural lipoma associated with a meningocele in a Manx-type cat. J Am Vet Med Assoc. (1993) 203:1159–61. 8244864

[10] Buckingham KJ McMillin MJ Brassil MM Shively KM Magnaye KM Cortes A. Multiple mutant T alleles cause haploinsufficiency of Brachyury and short tails in Manx cats. Mamm Genome. (2013) 24:400–8. 10.1007/s00335-013-9471-1 23949773 PMC3848309

[11] Haworth K Putt W Cattanach B Breen M Binns M Lingaas P. Canine homolog of the T-box transcription factor T; failure of the protein to bind to its DNA target leads to a short-tail phenotype. Mamm Genome. (2001) 12:212–8. 10.1007/s003350010253 11252170

[12] Hytönen MK Grall A Hédan B Dréano S Seguin SJ Delattre D. Ancestral T-box mutation is present in many, but not all, short-tailed dog breeds. J Hered. (2009) 100:236–40. 10.1093/jhered/esn085 18854372

[13] Amaral CB Romão MA Ferreira AM. Perosomus elumbis in a puppy. J Comp Pathol. (2012) 147:495–8. 10.1016/j.jcpa.2012.03.003 22578331

[14] Son JM Yong HY Lee DS Choi HJ Jeong SM Lee YW. A case of perosomus elumbis in a Holstein calf. J Vet Med Sc. (2008) 70:521–3. 10.1292/jvms.70.521 18525179

[15] Jagannathan V Drögemüller C Leeb T Dog Biomedical Variant Database Consortium (DBVDC). A comprehensive biomedical variant catalogue based on whole genome sequences of 582 dogs and eight wolves. Anim. Genet. (2019) 50:695–704. 10.1111/age.12834 31486122 PMC6842318

[16] Au KS Ashley-Koch A Northrup H. Epidemiologic and genetic aspects of spina bifida and other neural tube defects. Dev Disabil Res Rev. (2010) 16:6–15. 10.1002/ddrr.93 20419766 PMC3053142

[17] Thomson C Hahn C(editors). Neuroembriology. In: Veterinary Neuroanatomy: A Clinical Approach. St Louis: Saunders Elsevier Press (2012). p. 11–13.

[18] Martín Muñiz L Del Magno S Gandini G Pisoni L Menchetti M Foglia A. Surgical outcomes of six bulldogs with spinal lumbosacral meningomyelocele or meningocele. Vet Surg. (2020) 49:200–6. 10.1111/vsu.13342 31758707

[19] Bertram S Ter Haar G De Decker S. Congenital malformations of the lumbosacral vertebral column are common in neurologically normal French bulldogs, English bulldogs, and pugs, with breed-specific differences. Vet Radiol Ultrasound. (2019) 60:400–8. 10.1111/vru.12753 31050057

[20] Song RB Glass EN Kent M. Spina bifida, meningomyelocele, and meningocele. Vet Clin North Am Small Anim Pract. (2016) 46:327–45. 10.1016/j.cvsm.2015.10.007 26725976

[21] Balioglu MB Akman YE Ucpunar H Albayrak A Kargin D Atici Y. Sacral agenesis: evaluation of accompanying pathologies in 38 cases, with analysis of long-term outcomes. Childs Nerv Syst. (2016) 32:1693–702. 10.1007/s00381-016-3022-5 26872465

[22] Ladino Torres MF DiPietro MA. Spine ultrasound imaging in the newborn. Semin Ultrasound CT MR. (2014) 35:652–61. 10.1053/j.sult.2014.08.001 25454057

[23] Kylat RI Bader M. Caudal regression syndrome. Children. (2020) 7:211. 10.3390/children7110211 33158301 PMC7694368

[24] Singh SK Singh RD Sharma A. Caudal regression syndrome–case report and review of literature. Pediatr Surg Int. (2005) 21:578–81. 10.1007/s00383-005-1451-4 15977017

[25] Akhaddar A. Caudal regression syndrome (spinal thoraco-lumbo-sacro-coccygeal agenesis). World Neurosurg. (2020) 142:301–2. 10.1016/j.wneu.2020.07.055 32683002

[26] Dewberry L Peña A Mirsky D Ketzer J Bischoff A. Sacral agenesis and fecal incontinence: how to increase the index of suspicion. Pediatr Surg Int. (2019) 35:239–42. 10.1007/s00383-018-4402-6 30392128

[27] Macedo M Martins JL Freitas Filho LG. Sacral ratio and fecal continence in children with anorectal malformations. BJU Int. (2004) 94:893–4. 10.1111/j.1464-410X.2004.05053.x 15476529

[28] Garcia-Barceló MM Chi-Hang Lui V Miao X So MT Yuk-yu Leon T Yuan ZW. Mutational analysis of SHH and GLI3 in anorectal malformations. Birth Defects Res A Clin Mol Teratol. (2008) 82:644–8. 10.1002/bdra.20482 18655123

[29] Emans PJ Kootstra G Marcelis CL Beuls EA van Heurn LW. The Currarino triad: the variable expression. J Pediatr Surg. (2005) 40:1238–42. 10.1016/j.jpedsurg.2005.05.004 16080925

[30] Bevanda K MemidŽan I Boban-RaguŽ A. Caudal regression syndrome (Currarino syndrome) with chromosome mutation 9. Radiology Case Rep. (2020) 15:1184–8. 10.1016/j.radcr.2020.05.023 32550955 PMC7292899

[31] Martucciello G Torre M Belloni E Lerone M Pini Prato A Cama A. Currarino syndrome: proposal of a diagnostic and therapeutic protocol. J Pediatr Surg. (2004) 39:1305–11. 10.1016/j.jpedsurg.2004.05.003 15359381

[32] Dworschak GC Reutter HM Ludwig M. Currarino syndrome: a comprehensive genetic review of a rare congenital disorder. Orphanet J Rare Dis. (2021) 16:167. 10.1186/s13023-021-01799-0 33836786 PMC8034116

[33] Hybki GC Murphy LA Marchi JP Patlogar JE Brisson JO Nakamura RK. Lumbosacral agenesis in a cat. JFMS Open Rep. (2016) 2:2055116916628555. 10.1177/2055116916628555 28491410 PMC5362867

[34] Patrick T Gonzalez O Dick EJ Jr Kumar S. Perosomus elumbis in a stillborn rhesus macaque (Macaca mulatta): a case report. J Med Primatol. (2020) 49:110–2. 10.1111/jmp.12459 31912505 PMC7054159

[35] Avedillo LJ Camón J. Perosomus elumbis in a pig. Vet Rec. (2007) 160:127–9. 10.1136/vr.160.4.127 17259456

[36] Dennis SM. Perosomus elumbis in sheep. Aust Vet J. (1975) 51:135–6. 10.1111/j.1751-0813.1975.tb09436.x 1164284

[37] Jones CJ. Perosomus elumbis (vertebral agenesis and arthrogryposis) in a stillborn Holstein calf. Vet Pathol. (1999) 36:64–70. 10.1354/vp.36-1-64 9921758

[38] Balamurugan B Pridhavidhar ReddyYV, Jyothi K. Surgical management of dystocia due to Perosomus elumbis in a nondescript buffalo. J Entomol Zool Stud. (2018) 6:2472–4.

[39] Agerholm JS Holm W Schmidt M Hyttel P Fredholm M McEvoy FJ. Perosomus elumbis in Danish Holstein cattle. BMC Vet Res. (2014) 10:227. 10.1186/s12917-014-0227-2 25253618 PMC4181705

[40] Gerhauser I Geburek F Wohlsein P. Perosomus elumbis, cerebral aplasia, and spina bifida in an aborted thoroughbred foal. Res Vet Sci. (2012) 92:266–8. 10.1016/j.rvsc.2010.11.009 21146843

[41] Piegari G D'Anza E Costanza D Prisco F Meomartino L d'Aquino I. Perosomus elumbis in piglets: Pathological, radiological and cytogenetic findings. Animals. (2021) 11:1132. 10.3390/ani11041132 33921043 PMC8071472

[42] Gentile A Testoni S. Inherited disorders of cattle: a selected review. Slov Vet Res. (2006) 43:17–29.

[43] Ehlers K Stürje H Merker HJ Nau H. Valproic acid-induced spina bifida: a mouse model. Teratology. (1992) 45:145–54. 10.1002/tera.1420450208 1377411

[44] Shen ZY Zhang J Bai YZ Zhang SC. Diagnosis and management of fecal incontinence in children and adolescents. Front Pediatr. (2022) 10:1034240. 10.3389/fped.2022.1034240 36330370 PMC9623001

[45] Sinha S Shah MA Babu DM. Symptomatic lower urinary tract dysfunction in sacral agenesis: Potentially high risk? Indian J Urol. (2018) 34:56–61. 10.4103/iju.IJU_184_17 29343914 PMC5769251

[46] Siroky MB. Electromyography of the perineal floor. Urol Clin North Am. (1996) 23:299–307. 10.1016/S0094-0143(05)70312-8 8659028

